# Personally valued voices engage reward-motivated behaviour and brain responses

**DOI:** 10.1093/scan/nsaf056

**Published:** 2025-05-26

**Authors:** Elise Kanber, Jonathan P Roiser, Carolyn McGettigan

**Affiliations:** Speech, Hearing and Phonetic Sciences, UCL, 2 Wakefield Street, London WC1N 1PF, United Kingdom; Institute of Cognitive Neuroscience, UCL, 17 Queen Square, London WC1N 3AZ, United Kingdom; Speech, Hearing and Phonetic Sciences, UCL, 2 Wakefield Street, London WC1N 1PF, United Kingdom

**Keywords:** voice perception, voice identity, familiar voices, reward, motivation, fMRI

## Abstract

Humans often attach notions of value to hearing the voices of specific loved ones, yet there is sparse scientific evidence supporting these claims. We present three experiments—two behavioural and one neuroimaging functional magnetic resonance imaging (fMRI)—that tested whether personally-valued voices engage reward-motivated behaviour and associated brain responses. Using novel voice incentive delay tasks, we show that listeners respond faster in anticipation of hearing the speaking voice of their music idol than when anticipating an unfamiliar voice or a pure tone (Experiment 1). A second behavioural experiment indicated that familiarity alone was insufficient to engage stronger reward-motivated behaviour in comparison with an unfamiliar identity (Experiment 2). These behavioural patterns were further reflected in an fMRI experiment, where the idol voice condition most strongly engaged brain regions associated with reward processing while responses to other familiar and unfamiliar voice conditions were often equivalent (Experiment 3). Taken together, these studies provide evidence that voices can be effective rewards, in particular when they are associated with intense parasocial interest. Future research should determine whether these findings generalize to personally known individuals.

## Introduction

The human voice is a very powerful expression of individual identity, enabling the user to flexibly express their thoughts, moods, and intentions through verbal and nonverbal communication (Scott and McGettigan 2016, Lavan et al. 2019). For the listener, in turn, it is a signal that conveys information about the person who is speaking. Recent psychological investigations of voice identity perception have shown that first impressions of physical, personality, and social characteristics are formed quickly from voices, with strong inter-rater agreement (McAleer et al. 2014, Lavan 2022, Mileva and Lavan 2023). Other work has found that hearing a person’s voice, as opposed to merely reading their words, increases the perceived employability of a job candidate (Schroeder and Epley 2015), mitigates against the dehumanization of a political opponent (Schroeder et al. 2017), and fosters feelings of social connectedness (Kumar and Epley 2021). As the voices of strangers become, with experience, the voices of familiar celebrities, colleagues, friends, and partners, first impressions will change into responses that reflect our knowledge of those voices and their owners, as well as our affective responses to them (Kreiman and Sidtis 2011, Lavan and McGettigan 2023). However, the vast majority of research on the perception of familiar voices has focused on recognition and identification of these voices, rarely addressing their social, motivational, and affective impacts on the listener (Sidtis and Kreiman 2012, McGettigan 2015).

It has been proposed that voices, particularly those of personally-­relevant, familiar identities, may be rewarding stimuli (McGettigan 2015). Rewards are desired, appetitive, and positive outcomes of motivated behaviour that have the capacity to increase the frequency of that behaviour (Matyjek et al. 2020). Presentation of certain social stimuli (e.g. a thumbs up; a smiling face) has been shown to elicit reward-motivated behaviours such as greater speed, frequency, or physical effort of responses, and to engage specific brain networks associated in the anticipation, valuation, and learning of social rewards. The brain regions involved are widespread, including: a salience network [anterior insula (aIns) and anterior cingulate cortex (ACC); associated with reward anticipation]; the ventral striatum [nucleus accumbens (NAcc) and ventral tegmental area, both associated with encoding reward value]; ventromedial and orbital prefrontal cortices (vmPFC and OFC; associated with reward experience and learning); and the dorsal striatum (putamen and caudate nuclei; associated with decision-making and goal-directed action; see e.g. Martins et al. 2021).

There is evidence from face perception research that specific person identities, as examples of socially and affectively salient stimuli, are rewarding—this has been seen, e.g. via increased responses in brain areas strongly innervated by dopamine, such as the NAcc, when viewing a romantic partner’s face compared with unfamiliar faces (Aron et al. 2005, Acevedo et al. 2012). For voices, research has focused on the mother’s voice as a salient stimulus for children and adolescents. Studies have shown that the sound of the mother’s voice, rather than what is said, reduces salivary cortisol and increases oxytocin (which modulates reward-related brain circuitry) after a child is exposed to a stressor (Seltzer et al. 2010, 2012). Neuroimaging studies have shown greater activation in the salience network and mesolimbic reward circuitry (comprising the ventral striatum, vmPFC, and OFC) of children elicited by the mother’s voice compared with other female voices (Abrams et al. 2016), although with evidence that this profile flips towards a greater engagement by non-familiar voices in adolescence (Abrams et al. 2022).

The current study aimed to gain insights into the potential of specific voices to act as rewards for adult listeners. First, we sought to establish whether adult participants will engage in reward-motivated behaviour for voices. To do this, we employed a modification of the social incentive delay task (SID), which we refer to as the voice incentive delay (VID) task. In SID tasks, a visual cue informing participants of the possible reward outcome of a trial is followed, after an anticipatory delay, by a target stimulus (e.g. white square). If the participant responds quickly enough to the target, they receive the cued reward (or avoid a cued punishment). If they respond too slowly, the trial ends with either a neutral or an omitted outcome. In these studies, reaction times (RTs) are consistently inversely proportional to the reward magnitude (e.g. smile intensity), such that greater anticipated rewards elicit faster RTs, and the slowest reaction times are found for baseline/no-outcome conditions. The SID task has been used to examine various types of social rewards, from viewing positive, smiling faces (Delmonte et al. 2012, Cremers et al. 2014, Barman et al. 2015), to receiving spoken praise (e.g. ‘Good job!’; Dutra et al. 2015, Kollmann et al. 2017), written feedback (fast/slow; Kirsch et al. 2003), or other social approval (e.g. thumbs up, nodding; Gossen et al. 2014). SID tasks have also revealed anticipatory and consummatory brain responses associated with outcomes of varying value, where rewards of the greatest value show strongest engagement of regions including the ventral striatum (Spreckelmeyer et al. 2009, Rademacher et al. 2010, 2014).

In our VID tasks, we associated different visual cues with different audio outcome conditions, comprising vocal identities and non-vocal stimuli. For each participant, we modelled the highest level of vocal reward with the speaking voice of a world-famous pop star, of whom that participant self-identified as a ‘superfan’. We selected pop stars for the primary reason that musical performers are often associated with ‘superfan’ followings (e.g. Beatlemaniacs, Swifties, the Beyhive) and thus presented a more promising source of eligible participants—nonetheless, we used samples of speech rather than song for generalizability (most personality-relevant individuals are experienced auditorily through their speaking voice and not through song), and in order to isolate the experience of hearing the individual’s voice from the experience of hearing their creative product. We predicted that listeners would respond quicker to hear this ‘musical idol’ condition compared with other outcome conditions. Having established reward-motivated responses to the idol voice in two behavioural experiments, we then conducted a neuroimaging study to examine whether the neural underpinnings of the observed behaviour reflect the systems previously associated with reward anticipation and receipt of social stimuli. We predicted that the idol voice condition would elicit the strongest responses in these reward-related brain networks.

## Experiment 1

The first experiment focused on a defining feature of rewards: their ability to motivate behaviour. Although some previous SID studies have incorporated vocal stimuli, these were included as the medium for verbal feedback messages (e.g. ‘Good job!’). No previous study has, to our knowledge, used voice identity as an index of reward. We therefore employed a VID task with three outcome conditions: the musical idol, an unfamiliar voice, and a pure tone. Based on our prediction that the idol voice is more personally relevant and therefore more rewarding than the unfamiliar voice, and that human voice outcomes should be more socially salient than non-human sounds, we predicted differences in RTs between all three conditions, following the trend idol < unfamiliar voice < pure tone.

## Methods

### Participants

Participants (total *N* = 119: 96 female, 22 male, one undisclosed; mean age: 24.74 years; SD = 5.40; range: 18–40 years) were recruited. Nineteen participants who failed to respond quickly enough (i.e. before the target disappeared) in the VID task on more than 1/3 of trials were excluded, to ensure that participants had sufficient exposure to the three potential reward outcomes. This left a final sample of 100 participants (80 female, 19 male, 1 undisclosed; mean age: 24.5 years; SD: 10.31 years; range: 18–40 years) included in the subsequent data analysis. Participants were recruited via social media (including Twitter, Facebook, Instagram, and Reddit), with recruitment advertisements posted on fan pages for specific pop stars. The sample included participants who self-identified as ‘superfans’ of one of four pop stars pre-selected by the experimenter: Beyoncé (29 participants), Taylor Swift (37 participants), Justin Bieber (10 participants), and Harry Styles (24 participants). All participants had normal or corrected-to-normal vision and did not report any hearing difficulties. Due to the nature of the recruitment, it was not feasible to offer cash payments to all participants who completed the main study—they were instead given the option to enter into a prize draw for one of four Amazon vouchers (value totalling £100). Participants in pilot studies were paid at a rate of £7.50 per hour. Ethical approval was obtained via the UCL research ethics committee (approval code: SHaPS-2019-CM-030) and informed consent was provided by all participants.

An *a priori* power analysis was conducted based on results from the SID task reported by Rademacher et al. (2010), which estimated the effect size as d = 0.36 (df = 31, critical *t*-value (two-tailed) = 2.04; Cohen’s d = 2.04/sqrt(32)). Using G*Power (Faul et al. 2009) to test the difference between two dependent group means (paired) using a two-tailed test, with the effect size *d* = 0.36, and an alpha of .05 (two-tailed) showed that a total sample of 84 participants was required to achieve a power of 0.90. A sample of 100 was chosen to accommodate potential noise associated with online testing.

### Materials

Voice clips were taken from interviews found on YouTube (https://www.youtube.com/). Stimulus selection prioritized high audio quality and interviews that had been conducted in quiet (i.e. no background music or noise). Audio clips containing 1.5–2 seconds of meaningful (but non-identifying) speech from each target voice were extracted from the video clips using Praat (Boersma and Weenink 2005) and saved as mono WAV files. All stimuli were then RMS normed for amplitude and converted to MP3 format for use in the online testing platform (www.gorilla.sc; Anwyl-Irvine et al. 2020).

Final stimulus selection was informed by two pilot studies with independent groups of participants recruited from Prolific (www.prolific.co). The first study (*N* = 124 adult participants) was used to match each of the four idol voices (Beyoncé, etc) to a less familiar voice of similar pleasantness (i.e. an athlete of matched regional accent and apparent vocal gender). The second study selected the 24 clips to be used per voice in the VID task. See Supplemental Material for details of these pilot studies and Supplemental Appendix A for transcriptions of the speech content (generated using OpenAI’s Whisper: https://github.com/openai/whisper).

### Procedure

Participants completed the experiment online and remotely via Gorilla Experiment Builder (www.gorilla.sc; Anwyl-Irvine et al. 2020). The experiment was set up to only accept users on desktop or laptop computers and to reject participants on phones or tablets. Participants were informed via onscreen instructions that they would be listening to and making judgements about sounds and should therefore try to be in a quiet environment. All experimental tasks were explained via written onscreen instructions during the testing session.

The VID task consisted of a practice phase and a test phase. In the practice phase, participants were instructed to press the space key on their keyboard as fast as possible whenever an orange circle (target) appeared on the screen. On each of 45 trials, a cue symbol (circle with one horizontal line, circle with three horizontal lines, or a triangle) appeared onscreen for 250 ms, followed by a fixation cross for 500–1000 ms, and then the target (sized at 270x266 pixels), which remained onscreen until the space key was pressed. Mean RTs for each participant were calculated relative to the onscreen appearance of the target and used to define the duration *t* for which the target stayed onscreen in the VID task (i.e. a challenging, but not unattainable, threshold intended to motivate quick responses). An image of a loudspeaker icon was presented after the participant made a button press, with a short reminder that in the ‘real task’ participants should expect to hear sounds.

**Figure 1.**
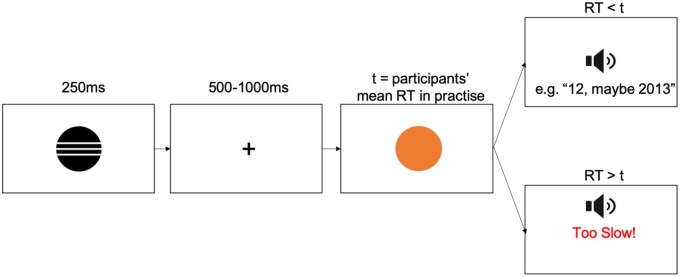
Trial structure for the voice incentive delay (VID) task.

Each of the 72 test trials in the VID task (24 trials × 3 cues) had the same structure as in the practice, with the exception that the target circle duration was individually defined, and the trial ended with presentation of an audio clip instead of a visual loudspeaker. Immediately prior to the task, participants were explicitly informed about which voice/sound each cue represented: circle with three horizontal lines = idol voice, circle with one line = athlete voice, triangle = pure tone. Thus, the cues either signalled potential reward (circles), or a non-voice outcome (triangle). If participants responded within the threshold *t*, they would hear the audio outcome associated with that trial’s cue; otherwise they heard the pure tone and saw the text ‘Too Slow!’ in red (see Fig. 1). Participants that responded too slowly on more than 1/3 of trials (24 trials) were not included in the subsequent analyses.

After the VID task, participants completed a series of validation tasks: (i) a recognition test of the cue-voice pairings, (ii) overall pleasantness ratings for the three outcome conditions (1–9 scale), (iii) a question about recognition of the athlete voice, and (iv) a multiple-choice quiz containing 10 questions about their chosen idol (e.g. ‘What is Beyoncé’s middle name?’; see Supplemental Material). Participants who responded incorrectly on the cue-voice pairing recognition test were not included in the final group analysis. The quiz was used to certify the fan status of the listeners but was not used to exclude any participants, as the different question sets were not calibrated for difficulty.

### Data analysis

Data were analysed with linear mixed effects models (LMMs) using the *lme4* package (Bates et al. 2015) in the R environment (R Core Team 2013). Model estimates and associated confidence intervals are reported as an estimate of the size of relevant effects. To assess the impact of the trial outcome on participants’ reaction times (RTs), an LMM was run with participant RT (ms) as the dependent variable, outcome as a fixed effect with three levels (musical idol, unfamiliar athlete, pure tone), and participant as a random intercept. All time-out trials (i.e. where RT > t) were excluded. Statistical significance was established via likelihood ratio tests comparing the full model that contained all fixed and random effects to a reduced model where the relevant effect (i.e. reward outcome) was dropped.

## Results and discussion

### Validation tasks

Two participants incorrectly matched the cues to the outcomes in the questionnaire task and were thus excluded, leaving 98 participants in the VID analysis.

A Friedman’s test with outcome type as the independent variable (three levels: idol voice, athlete voice, pure tone) and participant rating as the dependent variable found a significant main effect (*χ*2(2) = 171.41, *P* < .0001), where the musical idol was rated most pleasant (median = 9), followed by the athlete voice (median = 7), and the pure tone (median = 3). Paired Wilcoxon tests showed that all pairwise comparisons were significant (musical idol-athlete: *V *= 8628, *P* < .0001; musical idol-pure tone: *V *= 0474, *P* < .0001, athlete- tone: *V *= 8534, *P* < .0001).

One participant recognized the athlete by her profession (i.e. reported that she was a swimmer), but not by name, therefore, the participant was not excluded.

The majority of participants (85.7%) scored 7/10 or above in the multiple-choice quiz about their chosen idol. Although no participants were excluded based on quiz performance, the VID data were analysed with and without the participants who scored <7/10, and this did not change the results.

### VID task

For the analysis of RTs, timeout trials and trials with RTs faster than 150 ms were removed, leaving 4880 trials for inclusion in the linear mixed model analysis (participant means: 16.5 idol trials, 16.8 athlete trials, and 16.5 tone trials, respectively). Exploratory analyses on the full dataset (i.e. before participant and trial exclusions) showed that there was no effect of outcome type on the number of missed targets, indicating that participants were engaging with the task and achieving similarly accurate performance across all three conditions (see Supplemental Material).

**Figure 2.**
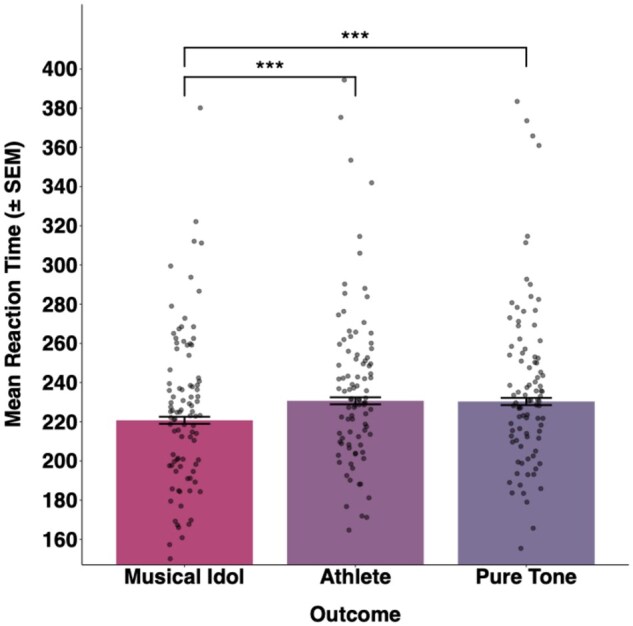
Bars display mean reaction times to the target in each outcome condition. Individual participants’ mean reaction times are displayed as individual points. Asterisks denote significance of post-hoc pairwise comparisons for reaction times between conditions. ***P < .0001.

Analysis of the final dataset showed that outcome type had a significant effect on RTs (*χ*^2^(2) = 38.74, *P* < .0001). Post-hoc comparisons with Bonferroni correction, run using *emmeans* (Lenth 2024), showed that participants were significantly faster when responding to the target following the idol cue (mean RT = 241.2 ms) compared to the athlete cue (mean RT = 246.9 ms, *t* = 5.39, *P* < .0001) and the pure tone cue (mean = 246.9 ms, *t* = 5.42, *P* < .0001). There was no significant difference in RTs for the unfamiliar athlete voice condition and the pure tone condition (*t* = 0.05, *P* = .96; see Fig. 2).

These results present two important findings: First, significantly quicker responses in anticipation of hearing a personally relevant voice (the idol voice) compared to an unfamiliar voice or a pure tone demonstrates that certain voice identities can act as larger rewards than others, similar to what has been previously shown for generic social rewards of differing intensities. Thus, we demonstrate that specific person identities can be differentially incentivizing as rewards in an incentive delay paradigm (cf studies comparing unfamiliar faces of varying attractiveness; e.g. Aharon et al. 2001). Further, the inclusion of four different Idol-Athlete pairings, carefully controlled for independent ratings of pleasantness, underscores the generalizability of the findings.

A second key finding is that we observe evidence for a dissociation in the patterns of ‘wanting’ (a reward’s motivational salience) versus ‘liking’ (its hedonic impact; Husain and Roiser 2018): while participants clearly differentiated the three outcome types when rating their perceived pleasantness, RTs were almost identical in the unfamiliar voice and the pure tone conditions. Aharon et al. (2001) similarly found that while heterosexual males rated both attractive male and female faces as more attractive than average faces, they only exerted greater effort to prolong viewing of attractive *female* faces. In the current study, it is also striking that the intrinsic motivational value of a human voice was no stronger than that for a simple tone. A previous study examined whether hearing unfamiliar voices of high versus low perceived trustworthiness and attractiveness elicited different trusting behaviours in an economic game, finding little-to-no effect (Knight et al. 2021). The current results add to these findings, suggesting that clear personal relevance—rather than enjoyment alone—may be necessary to engage motivated behaviour for voices.

## Experiment 2

The results of Experiment 1 provide important new evidence for the motivational qualities of human voice identities, suggesting that a personally-valued voice—in this case, the voice of the listener’s musical idol—is significantly more incentivizing than an unfamiliar voice or a tone. However, the design of Experiment 1 conflated familiarity with personal relevance, because the idol voice was the only identity that was recognizable to the participants. Thus, speeded RTs for the idol voice condition may have reflected motivation to hear a more familiar outcome, rather than a more valued one. We addressed this issue in Experiment 2, by creating a VID task with three voice identity outcomes: the idol voice, a familiar voice of lower personal relevance, and an unfamiliar voice. If familiarity explains the effects observed in Experiment 1, then RTs should be equivalent for the two familiar voice conditions and slower for the unfamiliar voice condition. If the effects were instead driven by personal relevance, RTs should be faster for the idol voice condition and equivalently slower for the familiar (non-idol) voice condition and the unfamiliar voice condition. To control for basic acoustic and perceptual differences between the familiar voice conditions, we selected two idol voices from Experiment 1—Taylor Swift and Beyoncé—and recruited participants who identified as superfans of one of these two artists. Thus, these two familiar voice identities always appeared in the task, in either the idol or familiar voice condition depending on the participant.

This study was run in conjunction with Experiment 3, which used functional magnetic resonance imaging (fMRI) to measure whether a VID task could engage similar brain responses to those seen in M/SID tasks.

## Methods

### Participants

Twenty-six participants (mean age = 22.88 years, SD = 4.97, age range = 18–39 years, 19 female) completed Experiment 2. Participants were recruited using a similar strategy as Experiment 1, including additional pre-screening for MRI eligibility. Twenty participants were superfans of Taylor Swift, and six participants were Beyoncé superfans. Ethical approval was obtained via the UCL research ethics committee (Approval code: fMRI/2019/005), and informed consent was given by all participants.

### Materials

Spontaneous speech excerpts (24 per voice) were extracted from YouTube interviews with two famous singers (Taylor Swift, Beyoncé) and a less familiar athlete (Allie Long; US soccer player). Voice excerpts were neutral (i.e. not expressive) and contained non-identifying speech content. All stimuli were saved as mono WAV files using PRAAT (Boersma and Weenink 2005). All stimuli were then RMS normed for amplitude and converted to MP3 format for use in the online testing platform (www.gorilla.sc; Anwyl-Irvine et al. 2020). Item durations ranged from 1.64 to 2.34 seconds (mean = 1.92, SD = 0.16 seconds). See Supplemental Appendix C for transcriptions of the voice stimuli.

### Procedure

The procedure for the practice phase was the same as Experiment 1 with the following exceptions: All visual stimuli were presented against a black background and the onscreen target was a white square (target). Each trial began with a cue shown for 250 ms, followed by a fixation cross for 500 ms, and a delay of 500–1000 ms (with blank screen) before the white square appeared.

After the practice, participants learned the associations between three cues and the three vocal identities: circle with three lines = musical idol (Beyoncé or Taylor), circle with two lines = familiar neutral celebrity (Taylor or Beyoncé), circle with one line = unfamiliar celebrity (Allie Long). In the main VID task (72 trials; 24 per cue), the target remained on the screen either until the space key was pressed, or until the time limit (set using the participant’s mean RT from the practice phase, as in Experiment 1) had been reached. Other event timings were as in the practice phase. Participants were informed that they would hear a short voice excerpt if they responded quickly enough, and if they did not hear anything they could assume that they were too slow.

### Data analysis

Reaction time data were analysed using a linear mixed model as for Experiment 1, including outcome condition as a fixed effect with three levels (musical idol, familiar, unfamiliar), and participant as a random intercept.

## Results and discussion

Due to a technical error, timeout thresholds were not properly applied within the VID task for two participants—this affected 2 test trials for one participant and 24 test trials for another, where the participants’ responses were slower than their practice phase threshold but the participant nonetheless heard the cued voice outcome instead of a silent outcome. Data from the more severely impacted participants were removed from further analysis. For the other participant, we removed the two missed timeout trials from the VID task data to ensure that only valid sub-threshold RT data were included in the group VID task analysis. Due to the overall limited sample size, no exclusion criterion was applied in terms of the overall number of timeouts in Experiment 2.

For the analysis of RTs, all timeout trials and trials with RTs faster than 150 ms were removed. This left 1030 trials for inclusion in the linear mixed model analysis (participant means: 15.1 idol trials, 13.6 familiar trials, and 13.0 unfamiliar trials, respectively).

**Figure 3.**
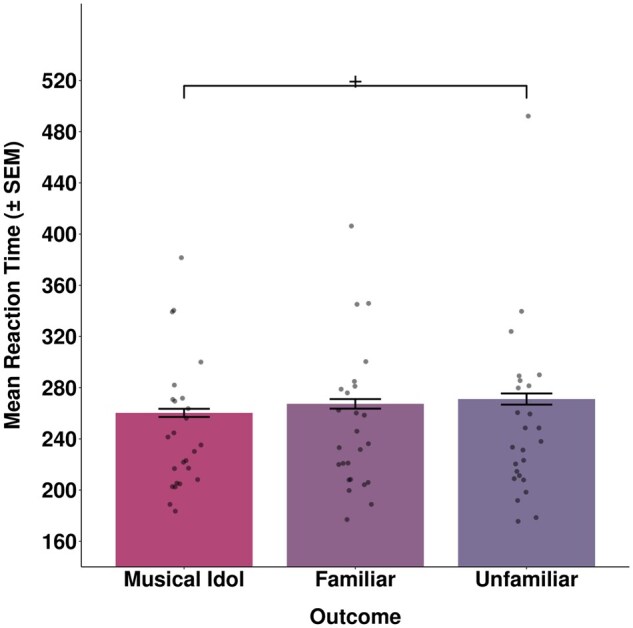
Bars display mean reaction times to the target in each outcome condition. Individual participants’ mean reaction times are displayed as individual points. Asterisks denote significance of post-hoc pairwise comparisons for reaction times between conditions. ^+^*P* < .05.

There was a significant effect of voice condition on participant reaction times (*χ*^2^(2) = 6.38, *P* = .041). Post-hoc pairwise comparisons using *emmeans* showed that participants were significantly faster to respond following the musical idol cue (mean RT = 260.3 ms) compared to the unfamiliar voice cue (mean RT = 271.1 ms; *t* = 2.51, *P* = .012). There was no significant difference in RTs between the musical idol condition and the familiar voice condition (mean RT = 267.3 ms; *t* = 1.44, *P* = .151), nor between the familiar voice condition and the unfamiliar voice condition (*t* = 1.07, *P* = .286; see Fig. 3).

The results indicate significantly greater motivation to hear the idol voice compared with an unfamiliar voice, as shown in Experiment 1. We also find tentative evidence that the increased motivation to hear the idol voice may be due to its greater personal relevance to the participants, rather than its familiarity alone. Specifically, the familiar non-idol voice condition showed statistically equivalent RTs to the unfamiliar voice condition, suggesting that familiarity alone does not increase motivation compared with our experimental baseline. However, we also found no significant difference between the idol and familiar conditions, suggesting that familiarity cannot be discounted as a mechanism for increased motivation to hear a voice.

Across two experiments, participants appeared to be equally motivated by simple tones, unfamiliar voices, and familiar but less personally relevant voices. The idol voice is distinguished as more ‘wanted’ as well as more ‘liked’ compared with unfamiliar voices and tones, although its distinction from other familiar voices is less clear. Future designs incorporating larger samples and multiple voices of personal relevance could test for more graded patterns of behaviour, e.g. by contrasting motivation in parasocial versus social relationships. For example, the parasocial connection of a ‘superfan’ to their idol and the early stages of romantic love could implicate more intensive seeking and appetitive behaviours than contexts in which a highly-valued voice is more readily available to the listener (e.g. long-term partner).

## Experiment 3

Having established that personally-valued voice identities can elicit reward-motivated behaviour in an incentive delay task, Experiment 3 sought to verify whether a VID task is underpinned by similar brain systems previously associated with social rewards in SID tasks (Martins et al. 2021). We therefore conducted an fMRI study with an in-scanner VID task pre-designed to include both ‘HIT’ trials that could result in an audio outcome, and ‘MISS’ trials that always resulted in no outcome. Thus, we created a 3 × 2 design in which we examined the effects of anticipating three different voice identities (i.e. main effect of identity), and the effects of reward receipt (versus absence) dependent on the identity being anticipated (i.e. interaction of identity and outcome).

Given the findings of Experiments 1 and 2, we expected to observe the strongest anticipatory and consummatory responses to the idol voice. Based on previous research examining brain responses in the SID task, we expected to see these responses in the salience network (aIns, ACC, thalamus, amygdala), as well as the ventral and dorsal striatum, vmPFC, OFC, posterior cingulate, and SMA (Martins et al. 2021). However, given the inclusion of familiar voices, we also expected to see brain regions previously associated with the perception of vocal stimuli (e.g. the temporal voice areas in bilateral STG/STS; Belin et al. 2000, Pernet et al. 2015), the processing of voice identities (right-dominant STS; Belin and Zatorre 2003, Kriegstein and Giraud 2004, Schall et al. 2015), as well as more general person perception (e.g. frontal pole, vmPFC/OFC, precuneus/posterior cingulate, temporal poles) and social processing [temporo-parietal junction (TPJ); Shah et al. 2001, Blank et al. 2014, Tsantani et al. 2019, Lally et al. 2023].

## Methods

### Participants

Twenty-five participants (mean age = 22.64 years, SD = 4.91, age range = 18–39 years, 19 female) completed the MRI session. This sample overlapped fully with the sample tested in Experiment 2; however, one Taylor Swift superfan was unable to complete the session due to a technical issue during MRI scanning.

### Materials

Spontaneous speech excerpts (42 per voice) were extracted from YouTube interviews with Taylor Swift, Beyoncé, and Allie Long. Voice excerpts were neutral (i.e. not expressive) and contained non-identifying speech content. All stimuli were saved as mono WAV files using PRAAT (Boersma and Weenink 2005) and RMS normed. Item durations ranged from 1.62 to 2.19 seconds (mean = 1.92, SD = 0.15 seconds). See Supplemental Appendix D for transcriptions of the voice stimuli.

### Procedure

Immediately following Experiment 2, participants were shown each of the three cue symbols from the VID task and asked to name the associated voice identity, to verify that they had learned the cue-voice pairings.

In the MRI scanner, auditory stimuli were presented via MR-compatible earphones (Sensimetrics Corporation, Woburn, MA), with sounds being played via MATLAB (version R2018b, Mathworks Inc., Natick, MA), using the Psychophysics Toolbox extension (http://psychtoolbox.org; Brainard 1997). Visual information was presented onscreen via a projector connected to a stimulus presentation laptop in the scanner control room. Participants viewed the screen via a mirror attached to the top of the head coil.

The in-scanner task had a 3x2 factorial design, with the factors voice identity (musical idol, familiar neutral celebrity, unfamiliar), and outcome (HIT, MISS). As in Experiment 2, the participant was instructed to respond (by pressing a button under their index finger) before the target (a white square) disappeared. In a HIT trial, an in-time response resulted in the immediate disappearance of the target, with audio playback of a voice excerpt as the trial outcome. In contrast, a MISS trial always ended with silence (i.e. no voice clip), regardless of the participant’s response time. To mitigate against participants recognizing the HIT/MISS manipulation and losing the sense that their actions affected the outcome, all null responses (i.e. where the participant did not press the button in time, or at all) were also followed by silence, regardless of whether the trial was predefined as a HIT.

**Figure 4.**
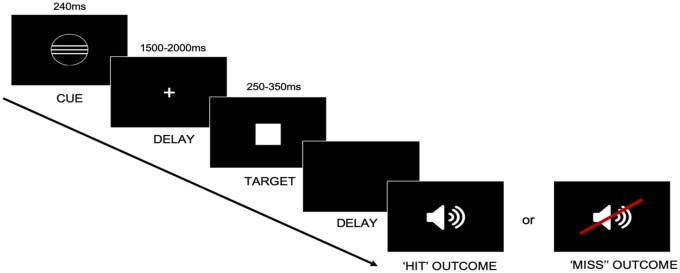
Experimental paradigm for the in-scanner social incentive delay task. Durations are displayed above each phase of the trial. On each trial, a cue provided information about the potential outcome participants could receive upon responding to a target (white square) within a set time window. The proportion of ‘HIT’ and ‘MISS’ outcomes was pre-set to 50% (where ‘HIT’ outcomes were still contingent on participant responses; see Methods).

Each trial began with the presentation of a cue for 240 ms, which signalled different possible trial outcomes (musical idol voice, familiar celebrity voice, unfamiliar voice). After a delay (jittered between 1500 and 2000 ms), a target was presented for between 250 and 350ms. For participants 1–13, the total time between the target appearing and the onset of the outcome was fixed at 350 ms (see Fig. 4), and all responses after the target disappeared were recorded as null. However, some participants struggled to respond in time, and thus heard fewer audio outcomes than anticipated. To preserve the number of HIT trials resulting in an audio outcome, the paradigm was adjusted for participant 14 onwards: the target still appeared for 250–350ms, but the permissible time window for responses was extended for 500 ms beyond the target’s disappearance.

The in-scanner task comprised 252 experimental trials (84 trials per run; 14 trials × 3 voice identities × 2 outcomes [HIT/MISS]). Voice excerpts were different in each functional run, meaning that participants maximally heard a total of 42 speech tokens from each of the three voices. Trial and stimulus order were fully randomized and different for each participant.

### MRI data acquisition

Scanning was performed on a 3 T MR scanner (MAGNETOM Prisma, Siemens, Erlangen, Germany), using a 32-channel head coil. Functional images were acquired over three runs, using an echo-planar imaging (EPI) sequence optimized for imaging the orbitofrontal cortex and amygdala (TR = 2.45 seconds, TA = 2.38 seconds, TE = 30 ms, flip- angle = 90 degrees, 35 slices, in-plane resolution = 3 mm × 3 mm × 2.5 mm, with an inter-slice gap of 0.5 mm, field-of-view = 192 mm; ascending acquisition). Field of view position was adjusted per participant to encompass the entirety of the frontal and temporal lobes, meaning slice positioning typically excluded the very top of the parietal lobes and the inferior half of the cerebellum. Five ‘dummy’ scans were presented immediately prior to the first trial of each run to allow for steady-state magnetization to become established; these were discarded and not included in the analyses. Each functional run lasted approximately 11 minutes. A whole-brain T1-weighted anatomical image was acquired between runs 2 and 3 (MPRAGE; 160 sagittal slices, voxel size = 1 mm isotropic).

### MRI data preprocessing and analysis

EPI data were pre-processed using SPM12 (Wellcome Centre for Human Neuroimaging, London, UK) implemented in MATLAB (R2018b, Mathworks Inc., Sherborn, MA, USA). Each participant’s functional images were realigned and corrected for slice-timing, co-registered with the anatomical image, spatially normalized and re-written into Montreal Neurological Institute stereotactic space with voxel dimensions 3 mm × 3 mm × 3 mm, and smoothed using a Gaussian kernel of 6 mm Full Width at Half Maximum (FWHM).

An event-related statistical analysis was performed in a two-level mixed-effects procedure. At the single subject level, a fixed-effects general linear model (GLM) modelled the onsets of the visual cue (i.e. the circle with 1, 2, or 3 lines presented at the beginning of each trial) for the six conditions (three voice conditions × two outcomes) as instantaneous events convolved with the canonical haemodynamic response function (HRF). By modelling only one event per trial, we intended to capture trial-wise BOLD responses per condition. In addition, the six rigid-body movement regressors (resulting from realignment) were included as regressors of no interest. Onsets for trials in which the participant made no response or a late response were not modelled.

For the group level random effects model, a 3x2 within-subjects ANOVA was conducted with voice condition (three levels: idol voice, familiar neutral celebrity, unfamiliar), and outcome (two levels: HIT/MISS) as within-subject factors, and using partitioned error approach (Henson and Penny 2003; see Supplemental Material for details). Whole-brain results are reported at an uncorrected voxelwise threshold of *P* < .001, with a cluster extent of k = 10 voxels; in Tables 1 and 2, we additionally highlight in clusters surviving whole-brain FWE cluster-level correction (*P* < .05) in SPM. Anatomical locations of peak voxels per cluster were determined using the Neuromorphometrics tool in SPM12. For plotting effects, mean parameter estimates per condition were extracted from each suprathreshold cluster, using the MarsBaR toolbox (Brett et al. 2002). These plots were further annotated to indicate the significance of post-hoc 2-tailed paired t-tests in R (Idol Hit vs. Familiar Hit; Idol Hit vs. Unfamiliar Hit; Familiar Hit vs. Unfamiliar Hit; Idol Miss vs. Familiar Miss; Idol Miss vs. Unfamiliar Miss; Familiar Miss vs. Unfamiliar Miss).

**Table 1.** Main effect of identity.

Contrast	Number of voxels (cluster)	*P* (cluster; FWE-corrected)	Anatomical label (peak)	Coordinate (peak)			*F* (peak)	*Z* (peak)	*P* (peak; FWE-corrected)
				x	y	z			
Main effect of identity	**399**	**<.001**	**Right anterior insula**	**36**	**17**	−**7**	**27.87**	**5.62**	**<.001**
	**414**	**<.001**	**Left IFG**	−**45**	**26**	−**1**	**22.99**	**5.20**	**.004**
	**80**	**<.001**	**Right STG/MTG**	**48**	−**37**	**2**	**18.21**	**4.70**	**.048**
	**18**	**.031**	**Right caudate**	**9**	**11**	**5**	**17.80**	**4.65**	**.061**
	**30**	**.002**	**Left cerebellum**	−**30**	−**58**	−**25**	**16.28**	**4.47**	**.149**
	**24**	**.007**	**Right supramarginal gyrus**	**57**	−**37**	**26**	**13.21**	**4.04**	**.816**
	15	.066	SMA	15	11	38	12.89	3.99	.864
	**26**	**.005**	**Middle cingulate gyrus**	−**3**	−**16**	**38**	**12.73**	**3.97**	**.886**
	**24**	**.007**	**SMA**	**6**	**20**	**56**	**12.53**	**3.93**	**.911**
	**35**	**.001**	**ACC**	−**6**	**35**	**8**	**12.23**	**3.89**	**.942**
	**17**	**.040**	**ACC**	**3**	**35**	**20**	**11.92**	**3.83**	**.966**
	10	.249	Left inferior occipital gyrus/occipital pole	−21	−97	−4	10.90	3.66	.997

Group contrast images were thresholded at voxel height *P* = .001 (uncorrected) and cluster extent *k* = 10. Coordinates are shown in Montreal Neurological Institute stereotactic space. Bold indicates clusters surviving FWE correction at *P* < .05.

**Table 2.** Interaction of outcome and identity.

Contrast	Number of voxels	*P* (cluster; FWE-corrected)	Anatomical label (peak)	Coordinate (peak)			*F* (peak)	*Z* (peak)	*P* (peak; FWE-corrected)
				x	y	z			
Interaction: outcome × identity	10	.245	Right STG/MTG	51	−31	2	15.94	4.42	.182
	10	.245	Left supramarginal gyrus, postcentral gyrus	−57	−25	29	14.14	4.18	.553
	**22**	**.011**	**Right supramarginal gyrus, postcentral gyrus**	**63**	−**19**	**35**	**13.22**	**4.04**	**.816**
	12	.143	Left supramarginal gyrus, parietal operculum	−63	−28	20	10.12	3.52	1

Group contrast images were thresholded at voxel height *P* = .001 (uncorrected) and cluster extent *k* = 10. Coordinates are shown in Montreal Neurological Institute stereotactic space. Bold indicates clusters surviving FWE correction at P < .05.

## Results and discussion

Our main interest was to inspect the effects of voice identity (Idol, Familiar voice, and Unfamiliar voice), independent of and dependent on trial outcome [i.e. hearing the voice (Hit) vs silence (Miss)]. Therefore, we only report the results of the Main Effect of Identity and the Interaction of Outcome × Identity in the manuscript. For the main effect of outcome, please see the Supplemental Materials.

### Main effect of identity

**Figure 5.**
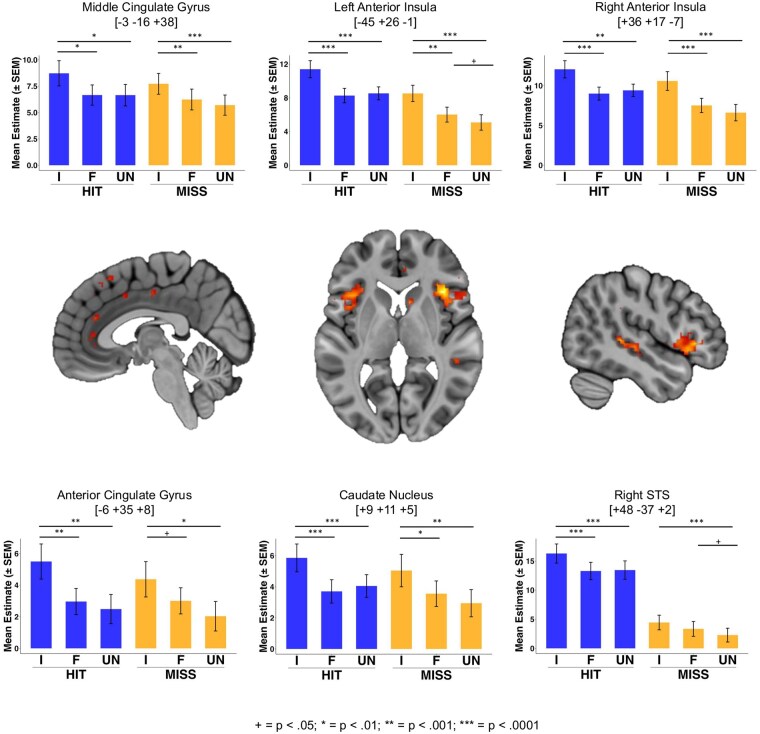
Brain regions showing a main effect of identity. Bar plots show mean parameter estimates per condition for each suprathreshold cluster.Peak voxels per cluster are indicated with coordinates in MNI space. Images are thresholded at a voxelwise threshold of *P* <.001, and a cluster extent of *k* = 10 voxels. I =Musical Idol, F = Familiar, UN = Unfamiliar. Annotations indicate the results of post-hoc pairwise comparisons: ^+^*P* < .05; *P < .01; **P < .001; ***P < .0001. See Table 2 for details of cluster and peak voxel statistics.

The main effect of identity revealed brain activations that were sensitive to the voice identity condition (Idol, Familiar, and Unfamiliar) regardless of the outcome of the trial. Therefore, we associate these activations primarily with differential anticipation of the three voice identities. Significant clusters (*P* < .05 FWE) were found in bilateral aIns/IFG, right STS, caudate nucleus, cerebellum, right SMG, left inferior occipital gyrus (IOG), and a range of medial sites along the cingulate gyrus and in supplementary motor area (SMA). All peaks showed an elevated response to the idol voice condition compared with both the familiar and unfamiliar voices; some peaks additionally showed evidence for a graded effect (Idol > Familiar > Unfamiliar) although with typically small differences between the familiar and unfamiliar conditions. See Fig. 5 and Table 1 for details of all significant clusters (*P* < .05 FWE) and other suprathreshold activations (voxel height threshold *P* < .001 uncorrected; cluster extent threshold 10 voxels).

**Figure 6.**
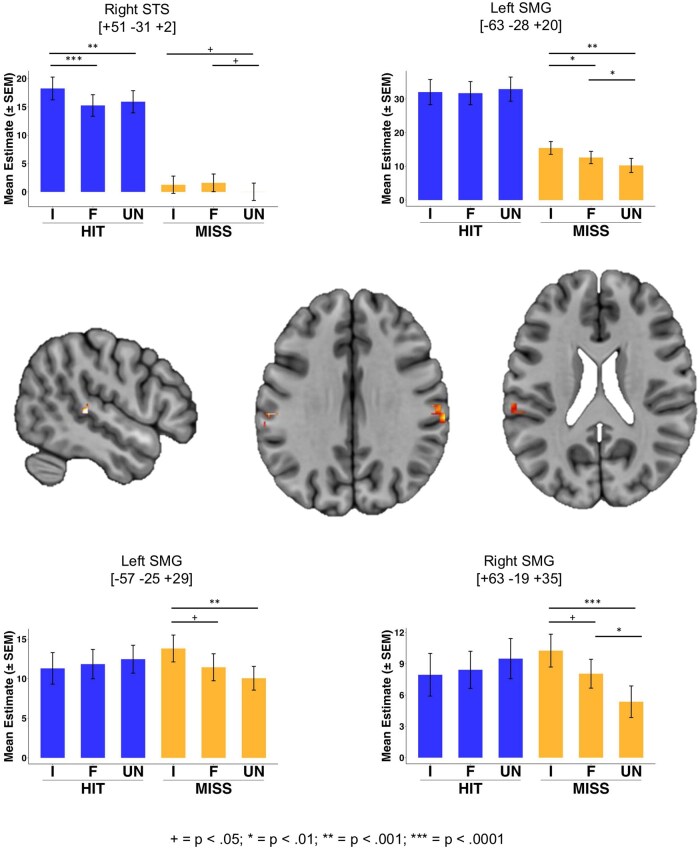
Brain regions showing an interaction of outcome and identity. Bar plots show mean parameter estimates per condition for each suprathreshold cluster. Peak voxels per cluster are indicated with coordinates in MNI space. Images are thresholded at a voxelwise threshold of *P* < .001, and a cluster extent of *k* = 10 voxels. I =Musical Idol, F = Familiar, UN = Unfamiliar. Annotations indicate the results of post-hoc pairwise comparisons: ^+^*P* < .05; *P< .01; ** = *P* < .001; ***P < .0001. See Table 3 for details of cluster and peak voxel statistics.

Within the suprathreshold clusters, voxels in left temporal pole, bilateral IFG, left aIns, and right STS showed overlap with the main effect of outcome (for the main effect of outcome, see Supplemental Material). Aside from these, the remaining effects, which showed no sensitivity to outcome (voice vs. silence), indicate a general prioritization of the idol identity regardless of the trial outcome and therefore likely relate more to reward anticipation than receipt/consumption.

The insula and anterior cingulate cortex are key components of a ‘salience network’ that has been implicated in reward anticipation, including within SID tasks (Martins et al. 2021), and to the sound of the mother’s voice in children (Abrams et al. 2019). Similarly, the caudate nucleus is implicated in the anticipation of social rewards and the avoidance of social punishments in SID tasks (Martins et al. 2021)—as part of the dorsal striatum, this region has been repeatedly implicated in social reward processing (Fareri and Delgado, 2014). Middle cingulate and SMA activations have also been associated with anticipation of social rewards in the SID and may reflect motor (and possibly cognitive) aspects of response readiness in line with the observed behaviour (i.e. faster responding in anticipation of the idol voice outcome).

### Interaction: outcome × identity

We observed one significant cluster (*P* < .05 FWE) in right supramarginal/postcentral gyrus (SMG/PoCG) showing an interaction of outcome and identity. This cluster, as well as two suprathreshold activations (voxel height threshold *P* < .001 uncorrected; cluster extent threshold 10 voxels) in left SMG indicate a similar profile, where responses to the three voice identities in MISS trials scaled with reward level (idol > familiar >/= unfamiliar) while there was no apparent trend in HIT trials. Martins et al. (2021) found decreased responses of bilateral SMG, with similar peak locations to those observed here, for trials in which listeners avoided social punishment. It’s important to highlight that in the current study, MISS trials generated no outcome *despite* participants responding in time, thus effects should not be explicable by differences in responding behaviours based on the outcome condition. Therefore, these small clusters may reflect some type of social punishment response.

Inspection of the parameter estimates from the other suprathreshold clusters points towards the right STS being primarily involved in responding to the voice outcome in HIT trials most strongly for the idol voice, while not being engaged in MISS trials (where there was no auditory outcome). The more inferior of the two left SMG activations also showed a stronger response for audio outcomes than silent outcomes. The STS, particularly in the right hemisphere, has been implicated in the processing of voice identities (Belin and Zatorre 2003, Kriegstein and Giraud 2004, Schall et al. 2015) and thus this activation may reflect a greater sensitivity of voice processing regions to the voice with which the participant likely had most prior experience (i.e. the Idol). See Fig. 6 and Table 2 for details of significant clusters and all other suprathreshold activations.

## General discussion

This study set out to test the proposal that individual voice identities could function as social rewards according to their personal relevance to the listener. Across three experiments, the accumulated evidence suggests that listeners were significantly more motivated to hear a personally-valued voice identity than to hear other familiar and unfamiliar voices and non-speech stimuli. This effect was seen in significantly speeded responses to the personally-valued voice in incentive delay tasks, as well as greater BOLD responses to this voice identity in brain regions associated with the anticipation and receipt of social rewards (Martins et al. 2021).

Strikingly, the behavioural effects observed here indicated no difference in reward value between an unfamiliar voice and a simple tone (Experiment 1), or between a familiar (but not personally-­valued) voice and an unfamiliar voice (Experiment 2), suggesting that it is the personal relevance that is the key attribute of the musical idol voices chosen for the current study that made them motivating to the listeners. However, there were indications of more graded effects: we did not find a difference between reaction times to the idol and familiar voices in Experiment 2 (although we acknowledge the limitation of a small sample due to the financial and eligibility constraints of the fMRI study for which the sample was recruited), and the brain responses in Experiment 3 also indicated some potential role for voice familiarity in reward-motivated behaviour alongside more consistent effects of personal value. It remains to be tested whether different levels of personal value and/or familiarity could dissociate these factors and delineate more graded profiles of reward value. First, understanding the apparent dissociation of ‘wanting’ from ‘liking’ for voices (Experiment 1) may benefit from designs including familiar identities that may be more liked than wanted—participants may experience intense positive affective experiences when hearing the voice of a close loved one with whom they speak daily (e.g. spouse, child), but they may be relatively more motivated by the opportunity to hear a voice that represents a more scarce resource (e.g. a celebrity). Second, to address the possibility that the idol voice in the current experiments was preferred due to being more valued *and* more familiar (as a result of the participants’ appetitive consumption of material relating to people they like), valence could be used as a means of more conclusively teasing apart value-related responses from familiarity—e.g. studies could compare responses to public figures of moderate familiarity but who are differentially appraised by the listener as positive (e.g. popular entertainer), neutral (e.g. newsreader), and negative (e.g. disliked politician). Interactions of value and familiarity are highly relevant to emerging voice technologies such as AI voice cloning, in which there may be trade-offs between the affective experience of a familiar voice and its perceived similarity to the human voice being replicated. Beyond the current paradigm, future work should also explore the wider implications of affective responses to specific voice identities. For example, enhancing audiobooks with personally-valued narrators may increase enjoyment, which has recently been shown to predict comprehension and onward motivation towards the written texts (see Bains et al. 2023). Other work could investigate how an elevated affective response to anticipating and hearing a specific voice identity (as has been demonstrated here) interacts with the perception of other cues in that voice, such as the acuity of appraising that speaker’s emotional states and the listeners’ own affective responses to these:

There are some additional points that warrant consideration for future work. In the current study, we exclusively used popular singers as the target personally-valued identities because we reasoned that ‘superfans’ are highly motivated by their musical idols in a broad sense, and therefore should value any opportunity to experience seeing, hearing and/or reading about these personalities beyond their musical performances/recordings alone. Crucially, we demonstrate the personal value of these voices empirically across our three experiments by showing how the idol voices were prioritized behaviourally and neurally over other audio stimuli. However, we used spoken stimuli throughout, and there is a chance that the observed effects would have been even stronger had we instead presented the participants with samples of the singing voice of their idols. Future work could test whether motivation for celebrity voices is dependent on the content (e.g. verbal vs. non-verbal) and modality (e.g. spoken vs. sung) of the audio stimuli, and further the extent to which this might depend on the familiarity of the audio content itself (e.g. excerpts from famous songs/movie scenes vs. previously unheard interview clips). Another consideration is that we used relatively neutral speech excerpts as the rewards in the current study (see Supplemental Appendixes A and C), but previous work has included materials ranging from explicit statements of support (e.g. Seltzer et al. 2010, 2012) to meaningless nonwords (e.g. Abrams et al. 2016, 2019, 2022). The voice, as it is typically encountered, conveys cues to identity, speech, and affective state in parallel (Belin et al. 2004)—a more systematic investigation combining different voice identities and spoken messages would reveal whether these factors have independent or interactive effects on the motivational and rewarding properties of the voice.

In summary, we demonstrate that adult listeners will assign reward differentially to specific human voice identities. We have an advanced understanding of reward for voices, specifically by moving beyond developmental populations and showing that a personally relevant voice motivates appetitive behaviour as well as engaging the brain’s reward and motivation circuitries. The paradigm is now apt to be tested for generalization to other person-specific stimuli, to test whether, e.g. faces and personal names might elicit similar effects.

## Supplementary Material

nsaf056_Supplementary_Data

## Data Availability

The thresholded group maps from the fMRI analysis, as well as the group mask (indicating the voxels included in group contrasts) are available via the Open Science Framework: https://osf.io/a7wev/.
