# Supplementary material for: Personally valued voices engage reward-motivated behaviour and brain responses
Source: Soc Cogn Affect Neurosci. 2025 May 26;20(1):nsaf056. doi: 10.1093/scan/nsaf056 (PMC12366989; doi:10.1093/scan/nsaf056)
Supplement: nsaf056_Supplementary_Data [file nsaf056_supplementary_data.docx]

**Supplemental Material**

**Experiment 1**

**Pilot Study 1**

This study was run to ensure that none of the singer voices was an extreme outlier in terms of overall pleasantness, and to match each idol voice to an unknown voice of similar pleasantness. For the unknown voices, three athletes were chosen per celebrity (12 athletes total). The chosen athletes were matched to the celebrities by broad regional accent and presumed gender. Independent raters (N=124) were recruited via Prolific.co (www.prolific.co), and each participant was randomly assigned to rate voice clips from one of the four singers (Justin Bieber: N = 32; Taylor Swift: N = 31; Beyoncé: N = 30; Harry Styles: N = 31) and their three matched athletes. Fifteen voice clips per speaker were included, making 60 stimuli in total. Participants rated each voice clip on “Pleasantness/Attractiveness”, “Valence”, and “Arousal” on a 9-point scale, where 1 represented very unattractive or unpleasant/negative/low arousal, and 9 represented very attractive or pleasant/positive/high arousal. For Pleasantness/Attractiveness, participants were asked: “How pleasant/attractive, or unpleasant/unattractive does the voice sound to you?” For Valence, participants were asked to rate how positive or negative the voice sounded. For Arousal, participants were asked: “How aroused does this sound to you? Low arousal: the sound is very drowsy and not energetic; High arousal: the sound is wakeful and energetic.” Participants rated each voice clip on the three traits by selecting a number with their mouse, and the order of the voice clips was fully randomised. Listeners were not told who the voices belonged to. Six catch trials were also included that required participants to select a specific number from 1-9 as specified by written text (e.g. “Please select the number 2”) to ensure sufficient attention was paid to the task. Participants were compensated at a rate of £7.50 per hour.

Mean pleasantness, valence, and arousal ratings were calculated for each of the 12 voices, and one athlete voice was selected to match each of the four celebrity voices, based primarily on pleasantness ratings. Ratings were similar and around the middle of the nine-point scale on average for pleasantness (mean = 5.48, range = 4.75 – 6.06) and valence (mean = 4.52, range = 4.17 – 6.06), with a slightly wider variation for arousal (mean = 5.19, range = 3.29 – 6.17; see Supplemental Table 1 for ratings for all tested voices).

**Supplemental Table 1.** Ratings of Pleasantness, Valence, and Arousal for each of the singer and athlete voices. The chosen celebrity-athlete pairs are arranged in adjacent rows and highlighted in bold. Standard deviations are reported in parentheses ().

| Speaker | Pleasantness |  | Valence |  | Arousal |
| --- | --- | --- | --- | --- | --- |
|  | *M SD* | *M* | *SD* | *M* | *SD* |
| **Taylor Swift** | **6.06 (1.03)** | **5.46** | **(0.76)** | **5.47** | **(1.04)** |
| **Julie Ertz** | **5.79 (0.71)** | **6.01** | **(0.77)** | **6.17** | **(0.88)** |
| Allie Long | 5.58 (0.92) | 5.22 | (0.69) | 5.14 | (0.80) |
| Emily Sonnett | 5.49 (1.02) | 5.22 | (0.69) | 5.15 | (0.84) |
| **Beyoncé** | **5.74 (0.98)** | **5.27** | **(0.79)** | **5.11** | **(0.76)** |
| **Simone Manuel** | **5.67 (0.97)** | **5.55** | **(0.73)** | **5.13** | **(0.74)** |
| Dominique Dawes | 5.79 (0.94) | 5.84 | (0.67) | 6.06 | (0.80) |
| Laila Ali | 5.32 (1.22) | 5.55 | (0.92) | 5.48 | (1.08) |
| **Justin Bieber** | **5.01 (1.34)** | **5.72** | **(0.84)** | **5.80** | **(0.94)** |
| **Sean Monahan** | **5.12 (1.64)** | **4.17** | **(1.03)** | **3.29** | **(1.18)** |
| Aaron Ekblad | 5.52 (1.11) | 5.55 | (0.80) | 5.03 | (0.87) |
| Tyson Jost | 5.53 (1.33) | 5.95 | (0.67) | 6.03 | (0.87) |
| **Harry Styles** | **5.76 (1.14)** | **5.05** | **(0.84)** | **4.35** | **(1.02)** |
| **Max Whitlock** | **5.33 (1.36)** | **6.06** | **(1.07)** | **5.77** | **(1.39)** |
| Nile Wilson | 5.25 (1.07) | 5.50 | (0.91) | 5.40 | (1.33) |
| Dan Crowley | 4.76 (1.16) | 4.68 | (1.01) | 3.80 | (1.23) |

**Pilot Study 2**

This study was run to ensure that the final stimuli chosen for the social incentive delay (SID) task were matched on overall pleasantness. Participants (N=120) were recruited on Prolific.co to rate 30 stimuli from one singer and 30 from their matched athlete voice (60 stimuli total) for Pleasantness, Valence, and Arousal, using 1-9 scales. Means and standard deviations were calculated for each of the voice clips, and for the overall ratings of each voice per trait rated. For each voice, 24 final clips were chosen that were rated around the middle of the pleasantness scale - any stimuli rated extremely high or low on any scale were discarded (see Supplemental Table 2 for descriptive statistics).

**Supplemental Table 2.** Ratings of pleasantness, valence, and arousal for each of the included celebrities and the matched athlete speakers, across the chosen 24 voice stimuli. Standard deviations are reported in parentheses.

| Speaker | Pleasantness | | Valence | | Arousal | |
| --- | --- | --- | --- | --- | --- | --- |
|  | *M* | *SD* | *M* | *SD* | *M* | *SD* |
| Beyoncé | 5.52 | (1.29) | 5.48 | (0.99) | 5.40 | (1.06) |
| Simone Manuel | 5.58 | (1.33) | 5.95 | (0.85) | 5.84 | (0.96) |
| Taylor Swift | 5.61 | (1.00) | 5.47 | (0.83) | 5.16 | (0.86) |
| Julie Ertz | 5.49 | (1.10) | 5.93 | (0.90) | 5.97 | (1.04) |
| Harry Styles | 5.35 | (1.23) | 4.65 | (0.83) | 4.02 | (1.11) |
| Max Whitlock | 4.96 | (1.40) | 5.87 | (0.89) | 5.99 | (1.09) |
| Justin Bieber | 5.99 | (1.34) | 5.84 | (1.00) | 5.63 | (1.05) |
| Sean Monahan | 5.75 | (1.54) | 4.88 | (1.01) | 3.75 | (1.25) |

The final pairs of celebrities and their matched athlete voices were as follows: Beyoncé & Simone Manuel, Taylor Swift & Julie Ertz, Harry Styles & Max Whitlock, and Justin Bieber & Sean Monahan. 192 voice clips in total were included in this experiment (24 clips x 4 celebrities + 24 clips x 4 athletes), as well as one pure tone (200Hz) generated using Audacity (https://audacityteam.org/). Voice excerpts were 2.0s in duration on average (range: 1.63 – 2.47 seconds), and the pure tone had a duration of 1.5s. The final set of stimuli were selected on the basis that they were rated “average” in terms of pleasantness, verified via ratings from an independent group of raters (see Stimulus Ratings 2 above).

**Quiz**

Appendix B shows the multiple-choice questions included for each musical idol. Participants were explicitly asked not to search for the answers to the quiz on the internet. After completing all ten questions, participants were invited to admit whether, and on which questions, they had cheated. Questions in which participants admitted to looking up the answers online were scored as incorrect, regardless of the answer. The majority of participants scored 7/10 or above (85.7% of participants). As the quiz questions were not standardised for difficulty across the four idols, therefore no participants were excluded on the basis of their accuracy scores. To validate this decision, the data were analysed with and without poorly scoring participants (i.e. those scoring <7/10), which did not change the results.

**Analysis of Missed Targets**

Missed targets were defined as trials for which participants did not respond with a button press to the target quicker than the predetermined threshold (set using the participants’ mean RT in a practice task). A binomial generalised linear mixed effects model (GLMM) was run with the binary variable hit/miss on each trial as the dependent measure, trial outcome (musical idol voice, athlete, pure tone) as a fixed effect, and participant as a random intercept. Statistical significance was established by comparing the full model that contained the fixed and random effect, to a reduced model that only contained the random effect. The comparison of the full to reduced model revealed that there was no statistically significant difference in the number of missed targets (χ^2^(2) = 5.86, p = .053) between the three outcomes (see Supplemental Figure 1).


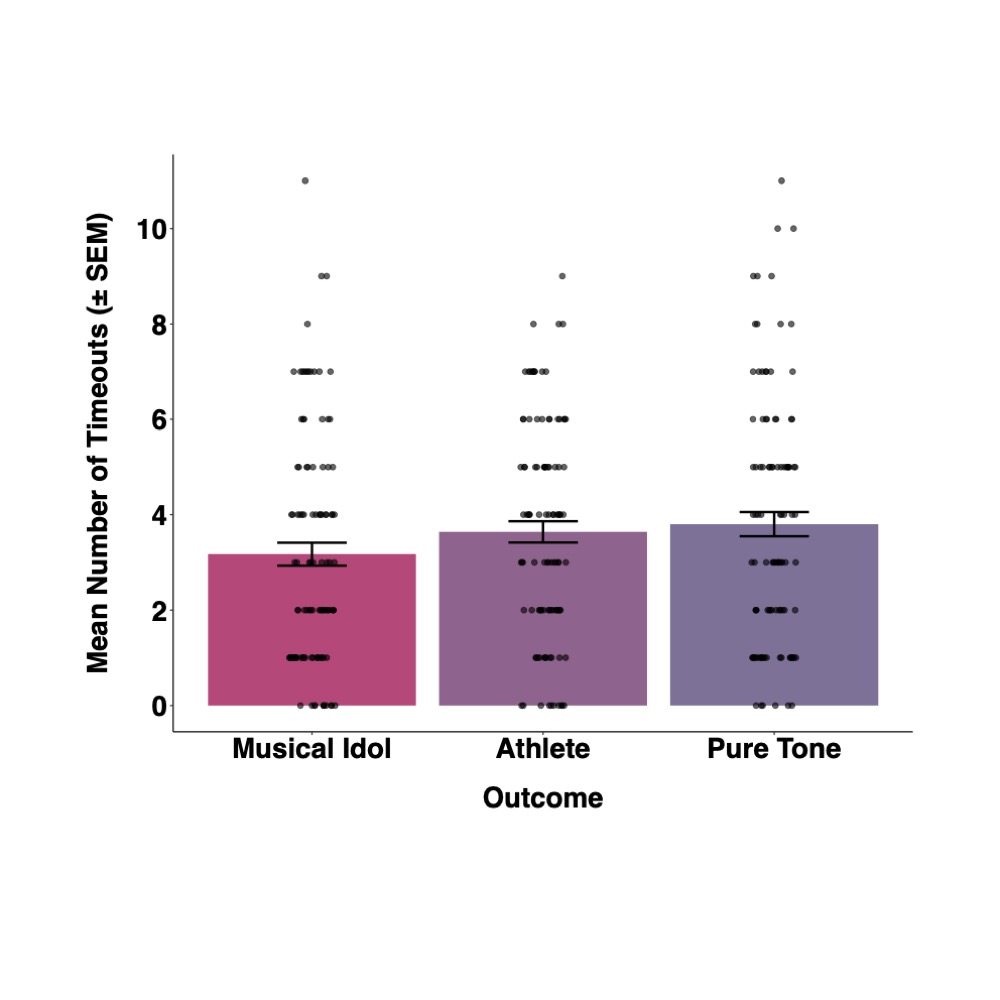


*Supplemental Figure 1. Bars display the mean number of missed targets across all participants for each outcome condition. Points display individual participant means.*

**Experiment 3**

**MRI data analysis**

For the group level random effects model, a 3x2 within-subjects ANOVA was conducted with voice condition (three levels: idol voice, familiar neutral celebrity, unfamiliar), and outcome (two levels: HIT/MISS) as within-subject factors, and using partitioned error approach (Henson & Penny, 2003). Under this approach, a set of differential effects is computed at the first level. Assuming the condition order [Idol_HIT_ Familiar_HIT_ Unfamiliar_HIT_ Idol_MISS_ Familiar_MISS_ Unfamiliar_MISS_] within each contrast, this corresponded to a first level T contrast of [1 1 1 -1 -1 -1] for the main effect of outcome (Hit > Miss), followed by a an F-test (with the contrast set to [1]) at the second level (testing for significant effects in both directions: Hit > Miss and Miss > Hit). To test for the main effect of identity (with three levels), differential effects were calculated at the first level, using three individual T contrasts [1 0 0 1 0 0], [0 1 0 0 1 0], and [0 0 1 0 0 1]. At the second level, these three contrasts are entered into a one-way within-subjects ANOVA using the 2-row F contrast [1 -1 0; 0 1 -1], to test for the main effect of identity. For the interaction, the differences of differential effects are calculated for each subject (Henson & Penny, 2003). We used the T contrasts [1 0 0 -1 0 0], [0 1 0 0 -1 0], [0 0 1 0 0 -1] at the first level. At the second level, these contrasts are entered into a one-way within-subjects ANOVA, using the 2-row F contrast [1 -1 0; 0 1 -1].

**Results & Discussion**

*Main effect of Outcome*

Significant clusters (p <. .05 FWE) showing greater responses during HIT than MISS trials were found in bilateral superior temporal lobes, the opercular part of the IFG in both hemispheres, as well as a number of sites in the brain’s midline including vmPFC and precuneus. Peaks in inferior occipital gyrus and left supramarginal/postcentral gyrus (SMG/PoCG) showed larger responses during MISS trials than HIT trials (Figure 5 and Table 1).

Engagement of the superior temporal lobes may reflect auditory/speech processing, as only HIT trials produced an audio outcome in the task. Temporal pole, vmPFC cortex, and precuneus are not typical in contrasts of speech over silence (which typically only engage perisylvian cortex), and here imply both (potentially common) processes of person identity perception (Tsantani et al., 2019; Blank et al., 2014) and social perception (Schilbach et al., 2012) and the receipt of social rewards (Martins et al., 2021; Fareri & Delgado, 2014). Overall, these findings suggest that the VID task employed here has indeed engaged social processes during reward receipt.

In their meta-analysis of social reward and punishment processing during SID tasks, Martins et al. (2021) reported increased BOLD responses in lateral occipital cortex when receiving a social reward; however the included studies often contrasted visually complex rewards such as faces and words with simpler neutral conditions including noise or meaningless symbols. One interpretation would be that these regions in lateral occipital cortex are engaged in the processing of onscreen visual objects (Grill-Spector et al., 2001): MISS trials in the current study will have been more quickly followed by new visual information on the screen (i.e. the cue to the next trial) than HIT trials (involving an auditory outcome of ~2 seconds in duration).


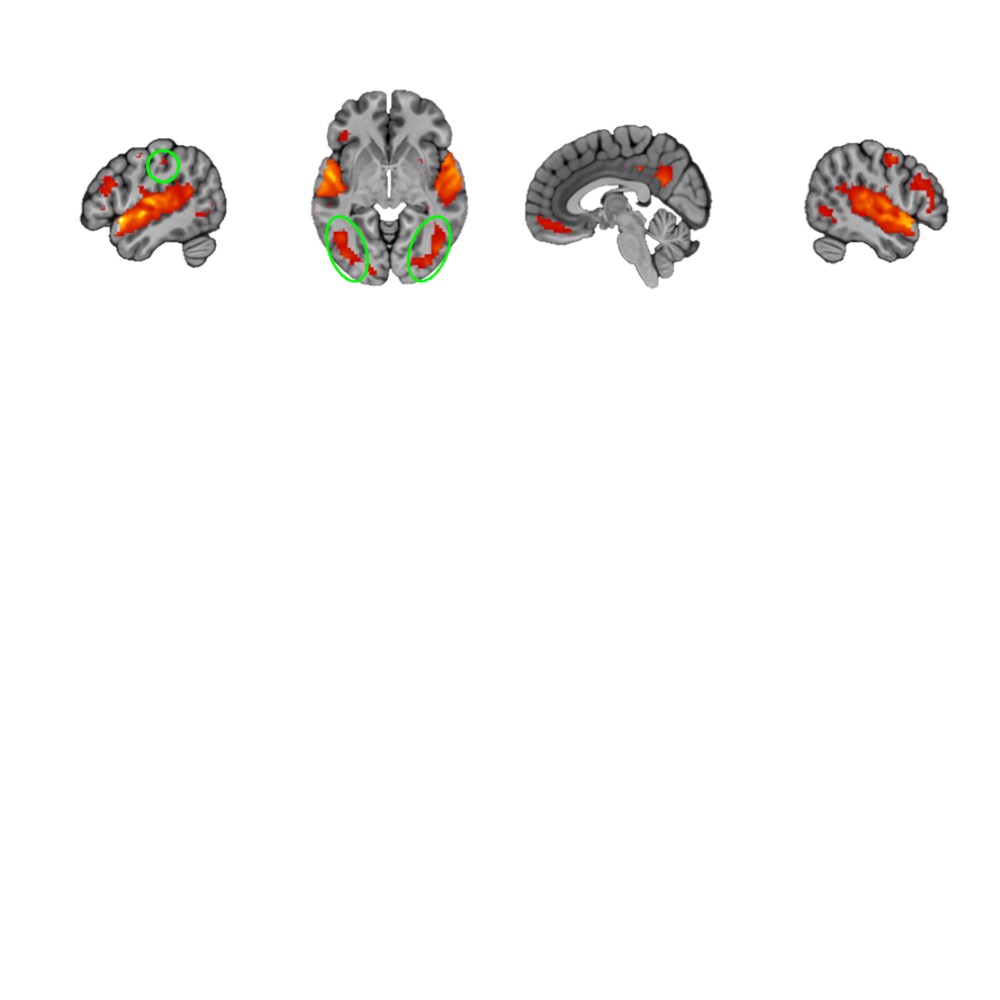


*Supplemental Figure 2. Brain regions showing a main effect of outcome (HIT compared with MISS). Circled clusters showed effects in the direction MISS > HIT. All other clusters showed effects in the direction HIT > MISS. Images are thresholded at a voxelwise threshold of p < .001, and a cluster extent of k = 10 voxels. See Table 1 for details of clusters and peak voxel statistics.*

**Supplemental References**

Grill-Spector, K., Kourtzi, Z., & Kanwisher, N. (2001). The lateral occipital complex and its role in object recognition. *Vision research*, *41*(10-11), 1409-1422.

Henson, R. N. A., & Penny, W. D. (2003). ANOVAs and SPM. Wellcome Department of Imaging Neuroscience, London, UK.

**Table 1: Main effect of outcome**

| **Contrast** | **Direction of effect** | **Number of voxels (cluster)** | **p  (cluster; FWE-corrected)** | **Anatomical Label (Peak)** | **Coordinate** | | | **F (peak)** | **z (peak)** | **p  (peak; FWE-corrected)** |
| --- | --- | --- | --- | --- | --- | --- | --- | --- | --- | --- |
|  |  |  |  |  | **x** | **y** | **z** |  |  |  |
| Main Effect of Outcome | Hit > Miss | **1766** | **<.001** | **Left temporal pole** | **-51** | **11** | **-10** | **222.38** | **7.32** | **<.001** |
|  |  | **1746** | **<.001** | **Right STG** | **60** | **-25** | **5** | **192.01** | **7.10** | **<.001** |
|  |  | 16 | .116 | Posterior cingulate gyrus | 3 | -34 | 35 | 77.87 | 5.72 | **<.001** |
|  |  | **137** | **<.001** | **Precuneus** | **3** | **-58** | **26** | **65.90** | **5.46** | **.001** |
|  |  | **73** | **<.001** | **Right precentral gyrus** | **54** | **-4** | **44** | **60.67** | **5.32** | **.002** |
|  |  | **182** | **<.001** | **Left IFG (pars opercularis)** | **-45** | **17** | **20** | **50.34** | **5.03** | **.009** |
|  |  | **306** | **<.001** | **Right IFG (pars opercularis)** | **57** | **23** | **23** | **50.11** | **5.02** | **.010** |
|  |  | 15 | .142 | Right hippocampus (white matter) | 36 | -31 | -7 | 38.44 | 4.60 | .078 |
|  |  | **154** | **<.001** | **Medial frontal gyrus** | **6** | **38** | **-19** | **37.37** | **4.56** | **.096** |
|  |  | **29** | **.01** | **Right IFG (pars orbitalis)** | **36** | **35** | **-16** | **36.30** | **4.51** | **.119** |
|  |  | 12 | .262 | Right middle occipital gyrus | 39 | 35 | -16 | 32.92 | 4.36 | .242 |
|  |  | **23** | **.029** | **Medial superior frontal gyrus** | **-6** | **50** | **35** | **31.09** | **4.27** | **.362** |
|  |  | 12 | .262 | Middle cingulate gyrus | -3 | -10 | 41 | 25.60 | 3.97 | .838 |
|  |  | **26** | **.017** | **Left precentral gyrus** | **-51** | **-7** | **50** | **24.47** | **3.90** | **.897** |
|  |  | 11 | .321 | Left cerebellum | -15 | -70 | -25 | 21.72 | 3.72 | .981 |
|  | Miss > Hit | **680** | **<.001** | **Right inferior occipital gyrus** | **42** | **-73** | **-4** | **75.05** | **5.66** | **<.001** |
|  |  | **558** | **<.001** | **Left inferior occipital gyrus** | **-42** | **-82** | **-4** | **69.66** | **5.54** | **.001** |
|  |  | **59** | **<.001** | **Left supramarginal gyrus, postcentral gyrus** | **-51** | **-28** | **44** | **23.50** | **3.84** | **.937** |

Group contrast images were thresholded at voxel height p = .001 (uncorrected) and cluster extent k = 10. Coordinates are shown in Montreal Neurological Institute stereotactic space. Bold indicates clusters surviving whole-brain FWE correction at p < .05.

**SUPPLEMENTAL APPENDIX A: Transcriptions of voice stimuli used in Experiment 1.**

| **Speaker** | **Clip** | **Transcription** |
| --- | --- | --- |
| Beyoncé Knowles | 1 | I always knew who I was. |
|  | 2 | You know, I'm learning that you can be kind. |
|  | 3 | I feel like that mystique is very important. |
|  | 4 | that I still just don't feel comfortable. |
|  | 5 | to tell my stories through photography. |
|  | 6 | That's what I chose to do. |
|  | 7 | and once you're aware of it. |
|  | 8 | your personal life as entertainment. |
|  | 9 | on a boat because then I have no excuse. |
|  | 10 | There's one thing when you're prepared, but when you don't know is... |
|  | 11 | It's been about seven years. |
|  | 12 | I mean, it's different for everyone. I was... |
|  | 13 | You know, when I traveled here... |
|  | 14 | and on television and... |
|  | 15 | I'm still kind of shocked. |
|  | 16 | and photographs and I've seen it. |
|  | 17 | I'm just so honored to be here and |
|  | 18 | Pray that one day I'd be able to do it. |
|  | 19 | It is all of the beautiful people. |
|  | 20 | and everything coming from the inside and everyone. |
|  | 21 | all the other things that are not as interesting and I... |
|  | 22 | People say that I'm a lot. |
|  | 23 | that you see all day every day. |
|  | 24 | you don't see the human form. |
| Harry Styles | 1 | I enjoyed all of them. |
|  | 2 | Be honest and connect to Gable. |
|  | 3 | probably like some of the saddest times of my life. |
|  | 4 | I never like taking them if I'm in like a... |
|  | 5 | Those were the first games that I watched. |
|  | 6 | 2012, maybe 2013. |
|  | 7 | I was told that the atmosphere was like... |
|  | 8 | They had like very big sausages. |
|  | 9 | cause a friend had us go. |
|  | 10 | and the really self-conscious about stuff. |
|  | 11 | you know how I define success. |
|  | 12 | through like conversations with friends. |
|  | 13 | let go of a lot of the strings. |
|  | 14 | There was a while where I was. |
|  | 15 | and that really stuck with me. |
|  | 16 | And I kind of thought about it. |
|  | 17 | rather than trying to keep everyone else happy. |
|  | 18 | It was written via like voice notes. |
|  | 19 | So that was kind of strange. It was like... |
|  | 20 | and kind of took it to a different... |
|  | 21 | You wanna hit well, that's how I feel about that. |
|  | 22 | That wasn't necessarily a bad thing. |
|  | 23 | and that it didn't mean it wasn't good. |
|  | 24 | It's gonna feel very free. |
| Julie Ertz | 1 | I feel great now like I had... |
|  | 2 | because it's just so cool to hear that. |
|  | 3 | So this time around I was like, you know what? |
|  | 4 | every moment. I really just try to soak in. |
|  | 5 | 2019 definitely has a special place in my heart. |
|  | 6 | and put myself in circumstances to be able to... |
|  | 7 | I will go back, my parents are making me go back. |
|  | 8 | we didn't really go on family vacation so that... |
|  | 9 | 2015 was amazing. I loved that |
|  | 10 | So different in so many ways. |
|  | 11 | It's been amazing and the support has been amazing. |
|  | 12 | I was like so excited, just been happy to be there. |
|  | 13 | I've gone through a lot in my career. |
|  | 14 | Everything I really just wanted to soak it all in. |
|  | 15 | but, to be honest, I didn't really know anything. |
|  | 16 | kind of that whole aspect in. |
|  | 17 | pushing boundaries of what it can be and... |
|  | 18 | be able to learn a little bit more about my journey. |
|  | 19 | and I was able to be challenged and pushed. |
|  | 20 | in a healthy way to be able to... |
|  | 21 | Just to like share it with my family was really cool. |
|  | 22 | This support has been amazing. I think obviously. |
|  | 23 | We're kind of in a bubble to really stay focused. |
|  | 24 | overwhelming in the great way. |
| Justin Bieber | 1 | I just really like expressing myself. |
|  | 2 | Yeah, it's her body and whatever she wants to do. |
|  | 3 | That was really what I was battling with. |
|  | 4 | and follow through with it, be a husband. |
|  | 5 | we all as humans get caught up in this. |
|  | 6 | We're all dealing with fear to some degree. |
|  | 7 | So I think at this point in my life... |
|  | 8 | It's been so busy, I'm just trying to get back into the gear. |
|  | 9 | I've been so chill for the past. I mean... |
|  | 10 | Am I ready for this? Am I ready to go back on the road? |
|  | 11 | I've been learning to just make the best of it and have fun. |
|  | 12 | Should I say this, should I say this, but... |
|  | 13 | And I'd done enough to be like, I don't really care. |
|  | 14 | like so when I snapped out of that. |
|  | 15 | so I kind of restarted my whole project. |
|  | 16 | and be passionate about what I love. |
|  | 17 | And last time I wasn't as hands on. |
|  | 18 | start with nothing and you come out with something which is |
|  | 19 | Basically for a while there I feel like I... |
|  | 20 | be encouraged by that and be inspired by that. |
|  | 21 | We have to quarantine for 15 days. |
|  | 22 | and I'm talking about stuff that I'm really going through. |
|  | 23 | To be honest, I made up a meaning. |
|  | 24 | Wait a month and if you still want to get it then get it. |
| Sean Monaghan | 1 | I couldn't be happier so where I want to be. |
|  | 2 | to be the best they can be. |
|  | 3 | That's definitely a relief to get that done. |
|  | 4 | I mean, I'm not worried that he's not gonna get done. |
|  | 5 | what he deserves and that's just a matter of time. |
|  | 6 | Yeah, for sure we've been talking about it. |
|  | 7 | No one really knows exactly how we're gonna do so it's... |
|  | 8 | It's a big commitment so I can thank... |
|  | 9 | That's a good feeling when you're playing on a team and... |
|  | 10 | Obviously I came in here at the young age and |
|  | 11 | He's still talking to guys every day and... |
|  | 12 | Yeah, I was, I was never concerned. |
|  | 13 | to come to agreement it's uh... |
|  | 14 | It was fair for both of us, so it's uh... |
|  | 15 | I think it's a good deal and... |
|  | 16 | Doesn't change anything to be honest. |
|  | 17 | Yeah, for sure, it takes time. |
|  | 18 | first contract we got to actually negotiate. |
|  | 19 | Positive is going to be here for... |
|  | 20 | feel like this is where I should be and |
|  | 21 | I had no doubt it was gonna get done and... |
|  | 22 | just a matter of time and now that it's done it's. |
|  | 23 | and I want to come in and have a great year. |
|  | 24 | This year is going to be real exciting. |
| Simone Manuel | 1 | The more I've been traveling, the more I realize that. |
|  | 2 | able to meet with her and kind of joke around with her. |
|  | 3 | She's good at Marco Polo. |
|  | 4 | I'm not sure what it's gonna be like. |
|  | 5 | Those moments we have to be when I'm talking to people. |
|  | 6 | so I'm really excited for that experience. |
|  | 7 | I think internally I'm really balancing it just... |
|  | 8 | And I love it and I enjoy it. |
|  | 9 | old model and someone you look up to and want to be like. |
|  | 10 | And the fact that now I'm that person to others. |
|  | 11 | I mean 70% of African Americans. |
|  | 12 | It's just inspiring that I can get somebody in. |
|  | 13 | can give so much back to someone else. And I think that's... |
|  | 14 | And that's really humbling to me because it's like... |
|  | 15 | I was rewarding about this whole experience. |
|  | 16 | I mean, they're talking to me in... |
|  | 17 | Hey, this is something I want to do. It's super cool. |
|  | 18 | I don't really know how to describe it. |
|  | 19 | You don't have any time to think about it. You just gotta go. |
|  | 20 | Oh wow, like I don't sit back and really... |
|  | 21 | It's been fun and I'm enjoying it all. |
|  | 22 | so much of an impact on other people. |
|  | 23 | that I just wrap them up in socks. |
|  | 24 | fuzzy sobs and keep them from scratching up. |
| Taylor Swift | 1 | the most emo dinner party. |
|  | 2 | we won't do this without this, I understand why. |
|  | 3 | and she speaks up for what she cares about. |
|  | 4 | more so than ever before, like. |
|  | 5 | definitely all about politics. |
|  | 6 | you just realize that it's part of the job. |
|  | 7 | Sarah, his amazing wife. |
|  | 8 | Do not get so caught up in this. |
|  | 9 | And so now when I see this happening. |
|  | 10 | people questioning whether I deserve to be there. |
|  | 11 | anatomically, biologically. |
|  | 12 | in other towns or cities or whatever. |
|  | 13 | something about the New York. |
|  | 14 | the night falls together like. |
|  | 15 | plan out exactly what you're gonna do. |
|  | 16 | end up somewhere else than you end up somewhere else. |
|  | 17 | I'm going to get my groceries. |
|  | 18 | It's been a really, really good time. |
|  | 19 | that makes it more special than anything I've ever done. |
|  | 20 | I choose to look now at the positive. |
|  | 21 | a very different way that I feel. |
|  | 22 | And it's hard to explain why that is. |
|  | 23 | between you and your previous work. |
|  | 24 | It was like something that... |
| Max Whitlock | 1 | That's one thing that me and Lea wanted, we wanted to... |
|  | 2 | Follow me around the world, get those experiences. |
|  | 3 | can't wait to revisit it back. |
|  | 4 | You know that it's coming round close, but um... |
|  | 5 | complete underdog as 19 years out. |
|  | 6 | I wasn't expected to produce any type of result. |
|  | 7 | good place to do it, I'll give it a shot. |
|  | 8 | which is a really tough job. |
|  | 9 | always my main focus. It always has been. |
|  | 10 | and it's finished, forget about it, let's move on. |
|  | 11 | I assume just that the plane is sort of a hit shift. |
|  | 12 | and you're just walking down the street, walking here. |
|  | 13 | This is incredible how many people have actually... |
|  | 14 | massive part of my success and um |
|  | 15 | She's been amazing. I just struggled a lot. |
|  | 16 | to do it for the first time for Majors. |
|  | 17 | after the back of 2018, you know. |
|  | 18 | I was taking Vix then, I was making upgrades. |
|  | 19 | Hopefully we could do the same job and hopefully we can boost it. |
|  | 20 | But me saying that then you have to go improve it. |
|  | 21 | I'm becoming a dad is the... |
|  | 22 | It makes your life busier, it makes everything else a little bit. |
|  | 23 | but for me to try and take pressure off as much as I can. |
|  | 24 | But if I try and think of it the same, try and do my job. |

**SUPPLEMENTAL APPENDIX B: Multiple-Choice Quizzes included in Experiment 1**

**Beyoncé**

1. What is Beyonce’s middle name?
   1. Solange
   2. Desiree
   3. Celestine
   4. Giselle
2. Beyoncé rose to fame in the late 1990s as the lead singer of what R&B girl-group?
   1. En Vogue
   2. Destiny’s Child
   3. The Spice Girls
   4. TLC
3. Beyoncé made her big screen debut in what movie?
   1. The Pink Panther
   2. Zoolander
   3. Dreamgirls
   4. Austin Powers in Goldmember
4. What was the name of Beyoncé's solo debut album?
   1. B’day
   2. Dangerously in Love
   3. I am … Sasha Fierce
   4. Survivor
5. In what song does Beyoncé sing: "I swore I'd never fall again, but this don't even feel like falling"?
   1. “Broken-hearted girl”
   2. “Halo”
   3. “Crazy in Love”
   4. “All night”
6. What is the name of Beyoncé's all-female tour band?
   1. Suga mama
   2. Parliament funkadelic
   3. Heartbreakers
   4. Sweet things
7. Who did Beyoncé marry in 2008?
   1. Jay-Z
   2. Sean Combs
   3. Ice Cube
   4. Kanye West
8. Destiny’s Child released their major label debut song on the soundtrack of what film?
   1. I, Robot
   2. Men in Black
   3. Enemy of the State
   4. Bad Boys
9. Who did Beyoncé portray in the movie *Cadillac Records*?
   1. Billie Holiday
   2. Aretha Franklin
   3. Etta James
   4. Ella Fitzgerald
10. What perfume did Beyoncé develop with Tommy Hilfiger?
    1. Enchanted
    2. Dreamgirl
    3. True star
    4. Truth or Dare

**Taylor Swift**

1. What was the title of Taylor’s first album?
   1. Taylor Swift
   2. Our Song
   3. Fearless
   4. Speak Now
2. What is Taylor Swift’s middle name?
   1. Elizabeth
   2. Renee
   3. Alison
   4. Sue
3. What famous rapper interrupted Taylor Swift’s speech at the 2009 VMAs?
   1. Jay-Z
   2. Kanye West
   3. Snoop Dogg
   4. Eminem
4. Who did Taylor write “We Are Never Ever Getting Back Together” about?
   1. Tom Hiddleston
   2. John Mayer
   3. Joe Jonas
   4. Jake Gyllenhaal
5. In what song does Taylor sing: “Cause the players gonna play, play, play, play, play”?
   1. “You belong with me”
   2. “Shake it off”
   3. “I knew you were trouble”
   4. “we are never ever getting back together”
6. Where did Taylor spend her early years?
   1. A missile silo
   2. A Christmas tree farm
   3. The biosphere
   4. An African safari
7. Taylor Swift met actor Taylor Lautner on the set of what movie?
   1. Twilight
   2. The Giver
   3. Valentine’s Day
   4. The Adventures of Sharkboy and Lavagirl
8. Which of Taylor’s songs earned her a Guinness World Record for fastest-selling digital single?
   1. “You belong with me”
   2. “We are never ever getting back together”
   3. “Shake it off”
   4. “Mine”
9. Taylor was the spokesperson for which NHL team?
   1. Flames
   2. Predators
   3. Kings
   4. Flyers
10. What was the lead single on Taylor Swift’s debut album?
    1. “Our song”
    2. “Picture to burn”
    3. “Tim McGraw”
    4. “Teardrops on my Guitar”

**Justin Bieber**

1. Where did Justin Bieber’s talent manager discover him?
   1. American Idol
   2. The Voice
   3. Star Search
   4. YouTube
2. What was the name of Justin’s debut album?
   1. My House
   2. My World
   3. My Life
   4. My Girl
3. On which popular TV show did Justin guest star in 2010?
   1. The Big Bang Theory
   2. CSI
   3. Pretty Little Liars
   4. Glee
4. What was Justin’s high school GPA?
   1. 1.97
   2. 4.0
   3. 2.35
   4. 3.84
5. What is Justin’s favourite food?
   1. Peanut Butter & Jelly
   2. Spaghetti
   3. Swedish fish
   4. Pizza
6. What song earned Justin his first Grammy Award?
   1. “Baby”
   2. “Purpose”
   3. “Where are Ü now”
   4. “Love Yourself”
7. Although Usher ultimately won out, what other singer wanted to mentor Justin?
   1. Michael Jackson
   2. Eminem
   3. Jay-Z
   4. Justin Timberlake
8. How many songs from Justin’s debut album made the Billboard Hot 100?

a. 3

b. 5

c. 7

d. 1

1. What was the name of Justin’s second studio album?
   1. Believe
   2. Purpose
   3. Urban Behavior
   4. Under the mistletoe
2. Justin serves as a celebrity spokesperson for what charity?
   1. Parliament of Promise
   2. Packs of Promise
   3. Pencils of Promise
   4. Projects of Promise

**Harry Styles**

1. What is Harry Styles’ middle name?
   1. William
   2. Edward
   3. Thomas
   4. Arthur
2. When is Harry’s birthday?
   1. March 23, 1994
   2. February 1, 1994
   3. February 28, 1993
   4. March 12, 1993
3. What is the name of Harry’s older sister?
   1. Gemma
   2. Poppy
   3. Claire
   4. Imogen
4. Harry made his film debut in which film?
   1. 1917
   2. Call Me by Your Name
   3. Dunkirk
   4. The Shape of Water
5. Which of these fruits is NOT featured in a Harry Styles song?
   1. Cherry
   2. Apple
   3. Kiwi
   4. Watermelon
6. What was the name of Harry’s debut solo tour?
   1. Harry Styles – Live on Tour
   2. Self-titled
   3. Treat People with Kindness
   4. Sign of the Tour
7. Which fictional island does the “Adore You” music video take place on?
   1. Narnia
   2. Eroda
   3. Avalon
   4. Nedlog
8. In which song does Harry sing “"And I'm well aware I write too many songs about you"?
   1. “Fine Line”
   2. “Cherry”
   3. “She”
   4. “Falling”
9. Which of the following songs was featured on Harry's FIRST album?
   1. “Little White Lies”
   2. “Ever Since New York”
   3. “She”
   4. “Seeing Blind”
10. What is the name of the bakery where Harry used to work as a teenager?
    1. W. Mandeville
    2. B. Warburton
    3. T. Maudsley
    4. J. Huntley

**SUPPLEMENTAL APPENDIX C: Transcriptions of voice stimuli used in Experiment 2.**

| **Speaker** | **Clip** | **Transcription** |
| --- | --- | --- |
| Allie Long | 1 | love being back in New York and |
|  | 2 | do what we feel is right and kind of... |
|  | 3 | you know, like people that are talking in... |
|  | 4 | So we need to be a team on this. |
|  | 5 | we need to come to an agreement. |
|  | 6 | Who's making the decision is like... |
|  | 7 | are lawyers and everyone. |
|  | 8 | and that night in between. |
|  | 9 | It's really cool and it's something that... |
|  | 10 | told me that I'll never make it. |
|  | 11 | I actually was like, I'm actually gonna prove you're all. |
|  | 12 | That probably happens everywhere and... |
|  | 13 | Unreal to be honest, it's honestly like |
|  | 14 | You work your whole life forward. |
|  | 15 | is so powerful and it's... |
|  | 16 | It's been a global inspiration. |
|  | 17 | when we got home we're like, wow. |
|  | 18 | and us as individuals and... |
|  | 19 | immediate or fast. |
|  | 20 | do it ever we can in our power. |
|  | 21 | It's a perfect example. We had people that... |
|  | 22 | All of it was for us. |
|  | 23 | It's just been so overwhelming. |
|  | 24 | I don't think we ever thought that this fight. |
| Taylor Swift | 1 | That's what I chose to do. |
|  | 2 | I am starting to live. |
|  | 3 | and once you're aware of it. |
|  | 4 | I feel like there's so many talented people. |
|  | 5 | I've grown a lot as a writer and... |
|  | 6 | talks about love and different relationships. |
|  | 7 | and the beats are a lot harder. |
|  | 8 | There's some really beautiful ballots. |
|  | 9 | I didn't actually get to work with her. |
|  | 10 | I talked to her on the phone and she was a really... |
|  | 11 | Hopefully, during another movie in September. |
|  | 12 | who I'm dating and who I'm not dating. |
|  | 13 | the media distorts things so much. |
|  | 14 | Every time I get in the car, I hear the song. |
|  | 15 | and on television and... |
|  | 16 | It's just the coolest festival. |
|  | 17 | I'm just so honored to be here and |
|  | 18 | Pray that one day I'd be able to do it. |
|  | 19 | It is all of the beautiful people. |
|  | 20 | all the other things that are not as interesting and I... |
|  | 21 | People say that I'm a lot. |
|  | 22 | and you've been doing this your whole life. |
|  | 23 | fearless and brave and... |
|  | 24 | It's an appropriate time to be a diva. |
| Beyoncé Knowles | 1 | the most emo dinner party. |
|  | 2 | we won't do this without this, I understand why. |
|  | 3 | and she speaks up for what she cares about. |
|  | 4 | more so than ever before, like. |
|  | 5 | definitely all about politics. |
|  | 6 | you just realize that it's part of the job. |
|  | 7 | Sarah, his amazing wife. |
|  | 8 | Do not get so caught up in this. |
|  | 9 | And so now when I see this happening. |
|  | 10 | people questioning whether I deserve to be there. |
|  | 11 | anatomically, biologically. |
|  | 12 | in other towns or cities or whatever. |
|  | 13 | something about the New York. |
|  | 14 | The night falls together like. |
|  | 15 | plan out exactly what you're gonna do. |
|  | 16 | End up somewhere else than you end up somewhere else. |
|  | 17 | I'm going to get my groceries. |
|  | 18 | It's been a really, really good time. |
|  | 19 | that makes it more special than anything I've ever done. |
|  | 20 | I choose to look now at the positive. |
|  | 21 | a very different way that I feel. |
|  | 22 | And it's hard to explain why that is. |
|  | 23 | between you and your previous work. |
|  | 24 | It was like something that... |

**SUPPLEMENTAL APPENDIX D: Transcriptions of voice stimuli used in Experiment 3.**

| **Speaker** | **Clip** | **Transcription** |
| --- | --- | --- |
| Allie Long | 1 | love being back in New York and |
|  | 2 | but it's something that we knew. |
|  | 3 | do what we feel is right and kind of... |
|  | 4 | doing this together on a unified front. |
|  | 5 | you know, like people that are talking in... |
|  | 6 | or cocky, we're arrogant, we're this. |
|  | 7 | the pool of money that is involved. |
|  | 8 | So we need to be a team on this. |
|  | 9 | we need to come to an agreement. |
|  | 10 | just shows that he actually believes in... |
|  | 11 | Who's making the decision is like... |
|  | 12 | are lawyers and everyone. |
|  | 13 | I haven't talked to too many, but I think that... |
|  | 14 | equality is like the only way. |
|  | 15 | and that night in between. |
|  | 16 | It's really cool and it's something that... |
|  | 17 | told me that I'll never make it. |
|  | 18 | So to actually win it is like |
|  | 19 | I had two options I could have either. |
|  | 20 | I actually was like, I'm actually gonna prove you're all. |
|  | 21 | and you can doubt me but... |
|  | 22 | What has made me who I am today? |
|  | 23 | That probably happens everywhere and... |
|  | 24 | Unreal to be honest, it's honestly like |
|  | 25 | for us as females too. |
|  | 26 | You work your whole life forward. |
|  | 27 | is so powerful and it's... |
|  | 28 | It's been a global inspiration. |
|  | 29 | when we got home we're like, wow. |
|  | 30 | that everything was shut down. |
|  | 31 | and us as individuals and... |
|  | 32 | immediate or fast. |
|  | 33 | that no matter how long it takes. |
|  | 34 | do it ever we can in our power. |
|  | 35 | bet on ourselves and and |
|  | 36 | attacking a lot of. |
|  | 37 | It's a perfect example. We had people that... |
|  | 38 | All of it was for us. |
|  | 39 | equality for women and |
|  | 40 | It's just been so overwhelming. |
|  | 41 | It's a responsibility that I feel like. |
|  | 42 | I don't think we ever thought that this fight. |
| Beyoncé Knowles | 1 | I always knew who I was. |
|  | 2 | push you as far as you allow them. |
|  | 3 | You know, I'm learning that you can be kind. |
|  | 4 | that I still just don't feel comfortable. |
|  | 5 | to tell my stories through photography. |
|  | 6 | That's what I chose to do. |
|  | 7 | I am starting to live. |
|  | 8 | and once you're aware of it. |
|  | 9 | I feel like there's so many talented people. |
|  | 10 | your personal life as entertainment. |
|  | 11 | I've grown a lot as a writer and... |
|  | 12 | talks about love and different relationships. |
|  | 13 | and the beats are a lot harder. |
|  | 14 | There's some really beautiful ballots. |
|  | 15 | I didn't actually get to work with her. |
|  | 16 | I talked to her on the phone and she was a really... |
|  | 17 | Hopefully, during another movie in September. |
|  | 18 | who I'm dating and who I'm not dating. |
|  | 19 | I think people are so brainwashed. |
|  | 20 | the media distorts things so much. |
|  | 21 | Every time I get in the car, I hear the song. |
|  | 22 | You know, when I traveled here... |
|  | 23 | and on television and... |
|  | 24 | I'm still kind of shocked. |
|  | 25 | and photographs and I've seen it. |
|  | 26 | It's just the coolest festival. |
|  | 27 | It was one of the most exciting nights. |
|  | 28 | I'm just so honored to be here and |
|  | 29 | Pray that one day I'd be able to do it. |
|  | 30 | You see all these pictures and... |
|  | 31 | It is all of the beautiful people. |
|  | 32 | and everything coming from the inside and everyone. |
|  | 33 | all the other things that are not as interesting and I... |
|  | 34 | People say that I'm a lot. |
|  | 35 | I was really, really afraid. |
|  | 36 | and you've been doing this your whole life. |
|  | 37 | talented and strong. |
|  | 38 | fearless and brave and... |
|  | 39 | All you think of is the picture. |
|  | 40 | It's an appropriate time to be a diva. |
|  | 41 | that you see all day every day. |
|  | 42 | you don't see the human form. |
| Taylor Swift | 1 | the most emo dinner party. |
|  | 2 | was almost a return to form. |
|  | 3 | I couldn't stop writing. |
|  | 4 | we won't do this without this, I understand why. |
|  | 5 | and she speaks up for what she cares about. |
|  | 6 | more so than ever before, like. |
|  | 7 | you just realize that it's part of the job. |
|  | 8 | when I'm kind of panicking and one of the things. |
|  | 9 | There's a lot of things that I tell myself. |
|  | 10 | Do not let anything stop you from making art. |
|  | 11 | Sarah, his amazing wife. |
|  | 12 | Do not get so caught up in this. |
|  | 13 | and succeeding at doing her job in making things. |
|  | 14 | And so now when I see this happening. |
|  | 15 | is somehow doing something wrong, but... |
|  | 16 | Women are not allowed to want those things. |
|  | 17 | So I feel like my whole career. |
|  | 18 | You watch them sensing things. |
|  | 19 | anatomically, biologically. |
|  | 20 | If they're still wanting to hang out, that's like... |
|  | 21 | I just got to ask them every... |
|  | 22 | I think my favorite thing about this city. |
|  | 23 | in other towns or cities or whatever. |
|  | 24 | something about the New York. |
|  | 25 | The night falls together like. |
|  | 26 | plan out exactly what you're gonna do. |
|  | 27 | End up somewhere else than you end up somewhere else. |
|  | 28 | I'm going to get my groceries. |
|  | 29 | I don't think you should ever have to apologize for your... |
|  | 30 | excitement just because it's like |
|  | 31 | doesn't mean that it's not something that's awesome. |
|  | 32 | if I had to pick a favorite lyricist. |
|  | 33 | It's been a really, really good time. |
|  | 34 | that makes it more special than anything I've ever done. |
|  | 35 | go back and revisit these moments of my life. |
|  | 36 | So it's actually really beautiful. |
|  | 37 | a very different way that I feel. |
|  | 38 | And it's hard to explain why that is. |
|  | 39 | It would be a tie between him and Lana Del Rey. |
|  | 40 | between you and your previous work. |
|  | 41 | but then it's compared to the first three and it... |
|  | 42 | It was like something that... |
